# Photoplethysmogram based vascular aging assessment using the deep convolutional neural network

**DOI:** 10.1038/s41598-022-15240-4

**Published:** 2022-07-05

**Authors:** Hangsik Shin, Gyujeong Noh, Byung-Moon Choi

**Affiliations:** 1 Department of Convergence Medicine, Asan Medical Center, University of Ulsan College of Medicine, Seoul, Republic of Korea; 2 Department of Anesthesiology and Pain Medicine, Asan Medical Center, University of Ulsan College of Medicine, Seoul, Republic of Korea; 3 Department of Clinical Pharmacology and Therapeutics, Asan Medical Center, University of Ulsan College of Medicine, Seoul, Republic of Korea

**Keywords:** Physical examination, Ageing

## Abstract

Arterial stiffness due to vascular aging is a major indicator during the assessment of cardiovascular risk. In this study, we propose a method for age estimation by applying deep learning to a photoplethysmogram (PPG) for the non-invasive assessment of the vascular age. The proposed deep learning-based age estimation model consists of three convolutional layers and two fully connected layers, and was developed as an explainable artificial intelligence model with Grad-Cam to explain the contribution of the PPG waveform characteristic to vascular age estimation. The deep learning model was developed using a segmented PPG by pulse from a total of 752 adults aged 20–89 years, and the performance was quantitatively evaluated using the mean absolute error, root-mean-squared-error, Pearson’s correlation coefficient, and coefficient of determination between the actual and estimated ages. As a result, a mean absolute error of 8.1 years, root mean squared error of 10.0 years, correlation coefficient of 0.61, and coefficient of determination of 0.37, were obtained. A Grad-Cam, used to determine the weight that the input signal contributes to the result, was employed to verify the contribution to the age estimation of the PPG segment, which was high around the systolic peak. The results of this study suggest that a convolutional-neural-network-based explainable artificial intelligence model outperforms existing models without an additional feature detection process. Moreover, it can provide a rationale for PPG-based vascular aging assessment.

## Introduction

Thomas Sydenham stated that, “A man is as old as his arteries.” As we age, the walls of arteries and arterioles thicken, the spaces within the arteries expand slightly, and the elastic tissue in the walls of arteries and arterioles is reduced. In addition, these changes make the blood vessels stiffer and less elastic. Therefore, arterial stiffness increases with vascular aging^1^. Arterial stiffness is a strong indicator of cardiovascular risk^2–6^ and asymptomatic individuals without overt cardiovascular disease^7,8^. Additionally, increased arterial stiffness is associated with risks of coronary heart disease, such as hypertension, diabetes mellitus, dyslipidemia, renal disease, and smoking^9^. The screening and management of cardiovascular (CV) risk is important to reduce the mortality, morbidity, and the socioeconomic burden associated with cardiovascular diseases (CVDs). The pulse wave velocity (PWV) is regarded as the clinical gold standard for the assessment of vascular aging. Experts regard the PWV as a simple, non-invasive, robust, and reproducible method for assessing arterial stiffness^10^. The normal and reference values for PWV were obtained from the Reference Value for Arterial Stiffness Collaboration using data gathered from 16,867 individuals and patients from 13 different centers across eight European countries^11^. The PWV can be measured in various ways. Among them, the carotid–femoral PWV (cfPWV), which is the velocity of transmission of the pulse wave along the arterial tree, is regarded as the best indicator to describe arterial stiffness^4,10–13^. The cfPWVs are usually obtained from waveforms at the right common carotid artery and right femoral artery, and the time delay is measured between the pulse onset of the two pulsatile waveforms. However, the cfPWVs may differ depending on the calculation method, since the cfPWV can be determined using different distances; either the direct carotid–femoral distance, or the subtracted distance (sternal to femoral—carotid to sternal). Consequently, the cfPWV can vary by up to 30% depending on the calculation method, and this difference is clinically significant^14,15^.

As such, for the PWV calculation, multi-site measurement of signals from various parts of the human body is required. This is difficult to apply in daily life because it is cumbersome to wear the equipment that it is used to record signals, not only on the fingers or wrists, but also on the ankles, thighs, or neck. In addition, it is necessary to consider the deviation due to the different distance calculation methods. To improve on this limitation, a technique for assessing vascular aging by analyzing the shape change of the photoplethysmogram (PPG) waveform measured at a single body site was proposed^16–21^. Since the PPG waveform can be modeled as a fluid flow change due to the incident wave-reflected wave relationship generated when a pulse-type pressure is applied to the tube (vessel)^22,23^, physical properties, such as compliance, distensibility, and stiffness of the tube, can be reflected in the PPG waveform. Based on these characteristics, the assessment of vascular stiffness using the original waveform or differential waveform of PPG and the main feature points has been attempted^24–26^. Takazawa derived aging-related indicators based on the second differential feature of PPG, and showed a correlation of 0.8 with the age^25^. In another study on hypertensive patients, the second-derivative PPG (SDPPG) aging index was considered a promising indicator, but compared to the PWV, the usefulness of the aging assessment was lower^24^. Millasseau developed a stiffness index (SI) and reflective index (RI) for estimating PWV based on the change in the PPG waveform due to vascular aging, and showed that SI had a correlation coefficient of 0.65 with the PWV, and a strong correlation with the age (R = 0.63)^16^. In addition, an index employing the features of PPG and second derivative PPG was developed to assess aging using PPG waveforms or feature points, where a correlation coefficient of 0.56 with the age was obtained^26^. Other studies have focused on the fact that the PPG is composed of a combination of incident and reflected wave components, including a study to find the correlation of the PPG with the age through the characteristics of the PPG reconstructed using multiple Gaussian distribution^20^, or Levenberg–Marquardt optimization algorithm (LMO)^18^. In a recent related study, a PPG was acquired using a smartphone camera and the age was estimated by applying machine-learning and deep-learning technology^21^, where an R^2^ ranging from 0.28 to 0.43 and RMSE ranging from 10.8 to 12.3 was obtained. Similar to the above study, various methods for estimating vascular aging using a single PPG have been proposed, however, continuous research on reliable methods is still required due to the insufficiency of subjects (N < 10)^18^, or lack of subject and measurement environment information^21^. Among the existing methods, several studies have reported on the usefulness of SDPPG, which has been evaluated for reproducibility^24–26^. However, the peak detection of the second-order differential PPG that is required to induce the SDPPG aging index is vulnerable to errors, and there are cases where the second-order differential feature point is not observed depending on the characteristics of the subject, thus, it is difficult to be utilized in everyday activities in typical environments^17,27,28^. Previous research results have suggested that PPG is a useful information-containing signal for the assessment of vascular aging^16–18,21,24–26,29–32^. Currently, most of the PPG-based vascular aging assessment studies have been performed by extracting the features of the PPG and estimating the age using a regression model^16,18,21,24–26,29^. Studies assessing vascular aging using detected features have shown a stable performance with the refined features. However, they have a limitation in that the cost of detecting features and making corrections for false detection is very high. Therefore, a method for applying deep learning to the PPG original waveform has recently been proposed^21,30–32^.

This study aims to assess vascular aging using the PPG to detect automatically optimized features by employing a deep learning-based approach without an empirical and manual feature detection process, and to evaluate the performance of the developed vascular aging evaluation model. In particular, this study uses Grad-Cam as an explainable artificial intelligence (XAI) to verify how the input PPG signal contributes to aging estimation. Since the deep learning-based approach is different, in that unlike the existing feature-based approach, the model automatically selects the optimal feature, there is a possibility that the estimation performance may be improved by using features that have not yet been discovered^33^. In addition, through the application of XAI, it is possible to trace the main waveform features in assessing vascular aging. Therefore, in the assessment of vascular aging using the PPG, an approach using deep learning, especially an XAI, can provide insight into key PPG characteristics as an indicator of vascular aging, as well as improve the assessment performance of vascular aging.

## Methods

### Study design and subjects

This study was designed as an observational study. The study protocols were approved (approval number: 2015 − 0104) and monitored by the institutional review board of the Asan Medical Center (Seoul, Republic of Korea), and informed consent to participate in the research studies was obtained from each subject before participating in the experiment. The entire research was performed in accordance with relevant guidelines and regulations. We enrolled patients aged 20 − 89 who were scheduled for elective surgery (thyroid, breast, or abdominal) between July and September 2015 at the Asan Medical Center, and had American Society of Anesthesiologists physical status of 1, 2, or 3. The exclusion criteria were as follows: women who were pregnant or lactating; clinically significant impairment of the cardiovascular, hepatic, or renal functions; history of cardiac arrhythmia; use of medication that might affect autonomic function; use of pre-operative analgesic or neuroleptic medication; and history of substance abuse or psychiatric disease. All patients were required to abstain from eating from midnight on the day of the surgery, and without any premedication. Patients were allowed to acclimatize for at least 5 min in the supine position in a quiet operating room with an ambient temperature ranging from 23 to 25℃. A specially designed sensor was placed between the columella and nasal septum to acquire a nasal photoplethysmography. The photoplethysmography data were collected for 3 min using E2-KIT pulse oximetry equipment (KTMED, Co. Ltd., Seoul, Korea) sampled with 125 or 250 Hz sampling frequency.

### Dataset

The photoplethysmography data were obtained from a total of 1,000 participants. Data for 752 of the participants were used for analysis, excluding data for 17 participants in which signal loss occurred due to device operation error, and 231 participants in which more than 50% of the section were not clear due to motion artifacts. The characteristics of the patients included in the analysis are summarized in Table 1.

**Table 1:** Characteristics of patients included in the analysis (N = 752).

Characteristic	Value
Male/female	331 (44.0)/421 (56.0)
ASA PS 1/2/3	465 (61.8)/ 253 (33.6)/ 35 (4.6)
Weight (kg)	60.1 (53.1–68.2)
Height (cm)	161.1 (155.8–167.2)
BMI (kg/m^2^)	23.1 (20.9–25.7)
Age (yr)	56 (46–65)
19–29	11 (1.3)
30–39	59 (7.9)
40–49	165 (21.9)
50–59	214 (28.5)
60–69	178 (23.7)
70–79	108 (14.4)
80–89	17 (2.3)
**Social characteristics**	
Smoking	109 (14.5)
Alcohol	217 (28.9)
**Medical history (multiple answers possible)**	
Hypertension	205 (27.3)
Diabetes mellitus	71 (9.4)
Pulmonary disease	17 (2.3)
Renal disease	6 (0.8)
Hepatic disease	24 (3.2)
Neurologic disease	5 (0.7)
Others	3 (0.4)

Data are presented as counts (percent) or median (25–75) where appropriate. ASA PS: American Society of Anesthesiologists Physical Status (1: a normal healthy patient, 2: a patient with mild systemic disease, 3: a patient with severe systemic disease), BMI: body mass index; Pulmonary disease: asthma (10), emphysema (2), bronchiectasis (1), chronic obstructive pulmonary disease (3), old tuberculosis (1). Renal disease: chronic kidney disease (2), end stage renal disease (4), Hepatic disease: hepatitis B virus (12), hepatitis C virus (4), liver cirrhosis (8), Neurologic disease: stroke (1). Cardiovascular accident (3), Others: angina (1), carotid artery stenosis (1), myelodysplastic syndrome (1).

### Data preprocessing

**Figure 1:**
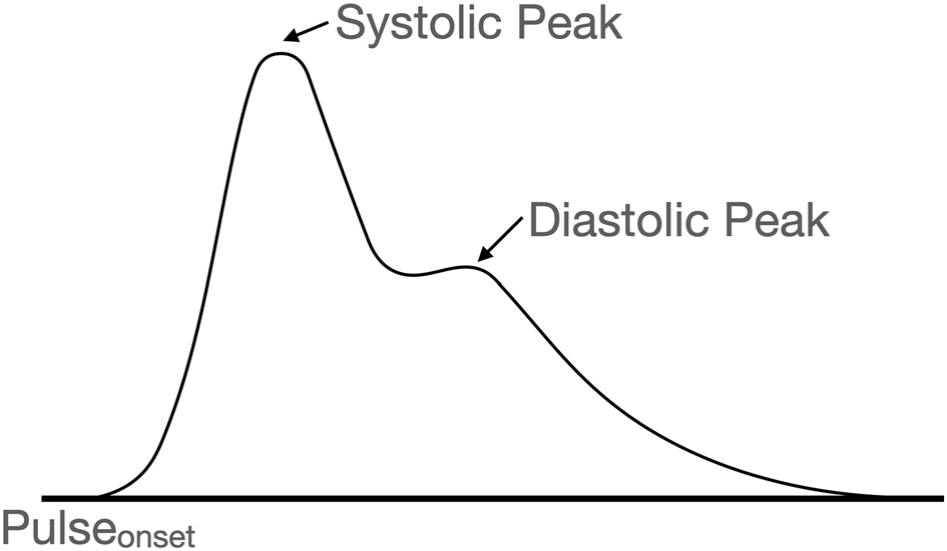
PPG segment and feature points.

**Figure 2:**
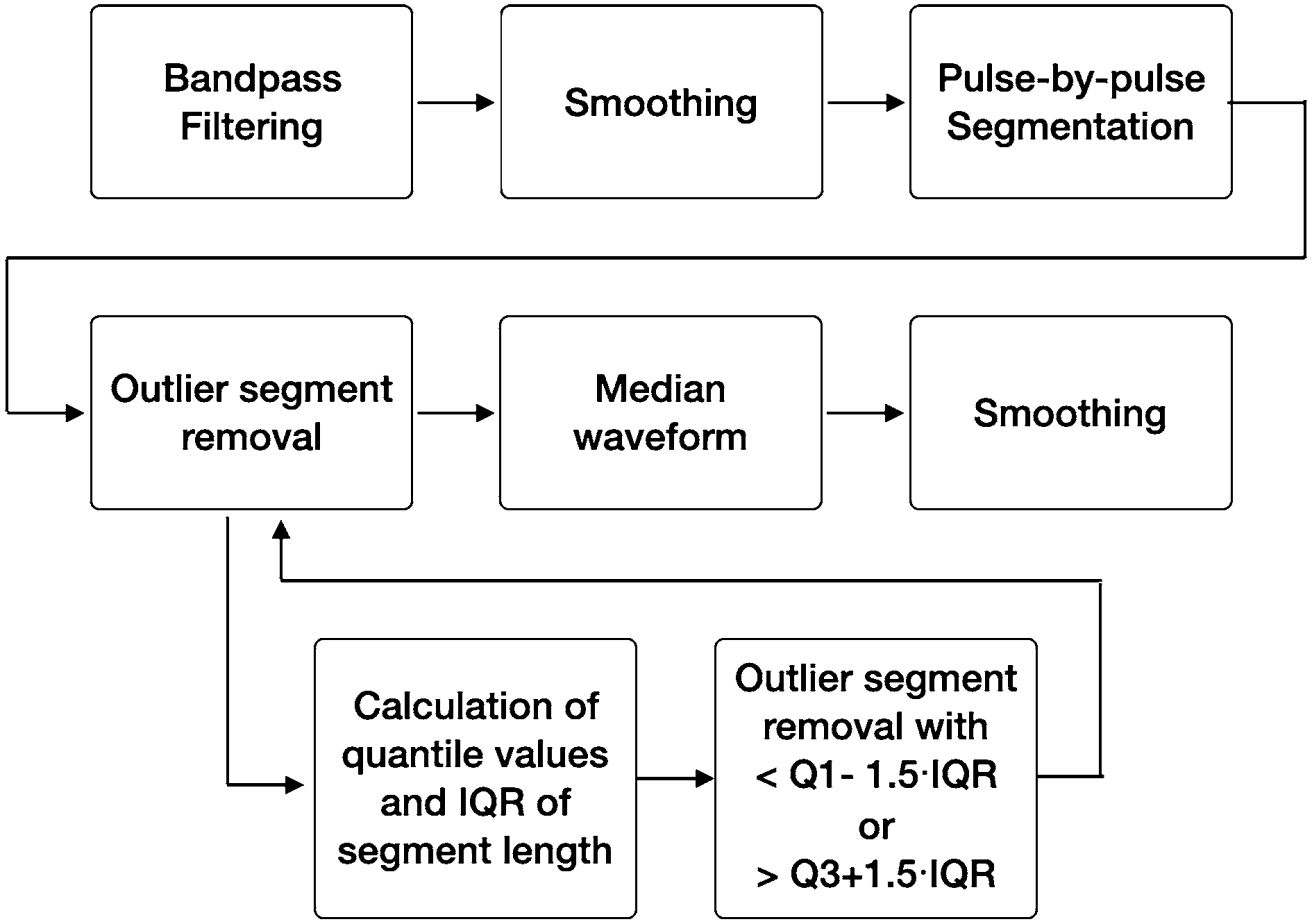
Data preprocessing procedures for deriving the representative photoplethysmogram pulse. The bandpass filter is a finite impulse response filter with a 0.5–10 Hz passband. Smoothening of the signal was performed using a moving average filter with a 50 ms window length. IQR: inter-quantile range.

In this study, a representative PPG pulsation waveform was generated and utilized for the identification of vascular aging. The representative PPG pulsation waveform generation process is as follows: First, the effect of breathing or high-frequency noise was removed by using a finite impulse response (FIR) bandpass filter. The FIR filter was designed to have a 0.5–10 Hz passband, and to have a number of taps equal to 125 or 250, depending on the sampling frequency. Subsequently, a moving averaging filter with a 50 ms window length was applied to smoothen the signal. Next, to obtain the individual PPG pulses, the PPG was segmented based on the pulse onset (Pulse_onset_), which is an inflection point that has a minimum value between pulses. Figure 1 shows a PPG segment and its representative features. The adaptive threshold peak detection method^34^ was used for pulse onset detection. In the case of the automatic segmentation of the PPG, a segmentation error may occur due to false pulse onset detection. Therefore, to minimize segmentation errors, segments in the normal range were selected based on the pulse-to-pulse interval (PPI). For normal segment identification, the pulse-to-pulse interval based on pulse onset was used. In this process, pulse segmentation was first performed, and only the segments corresponding to the normal range of the PPI were selected, excluding the PPI outliers. The PPI outlier was defined as a value smaller than Q1 − 1.5 × QD, or greater than Q3 + 1.5 × QD, by applying the 1.5 inter-quantile range (IQR) criterion; Q1: 1st quantile, Q3: 3rd quantile, and QD: quantile deviation. Subsequently, representative PPG pulses were obtained using only segments where the segment length did not correspond to the outlier determination criteria. The representative PPG pulses were then obtained by taking the median of the PPG segment at each sample location. At this time, since the length of each PPG segment may be different, each segment was interpolated to have a median length of the PPI, and cubic spline interpolation was used for segment interpolation. Finally, a representative waveform was generated by applying a moving average filter with a 50 ms window length to the waveform obtained from the median. Figure 2 describes the data processing process. For the input of the deep learning model, the representative waveform was normalized by scaling it to between 0 and 1, after which it was resized according to the size of the input vector (144 samples). At this time, resizing was performed using the Matlab 2020b function (The MathWorks, Inc., MA, USA), and resampling and normalization was performed with a value in the range of 0–1.

### Deep learning model

**Figure 3:**
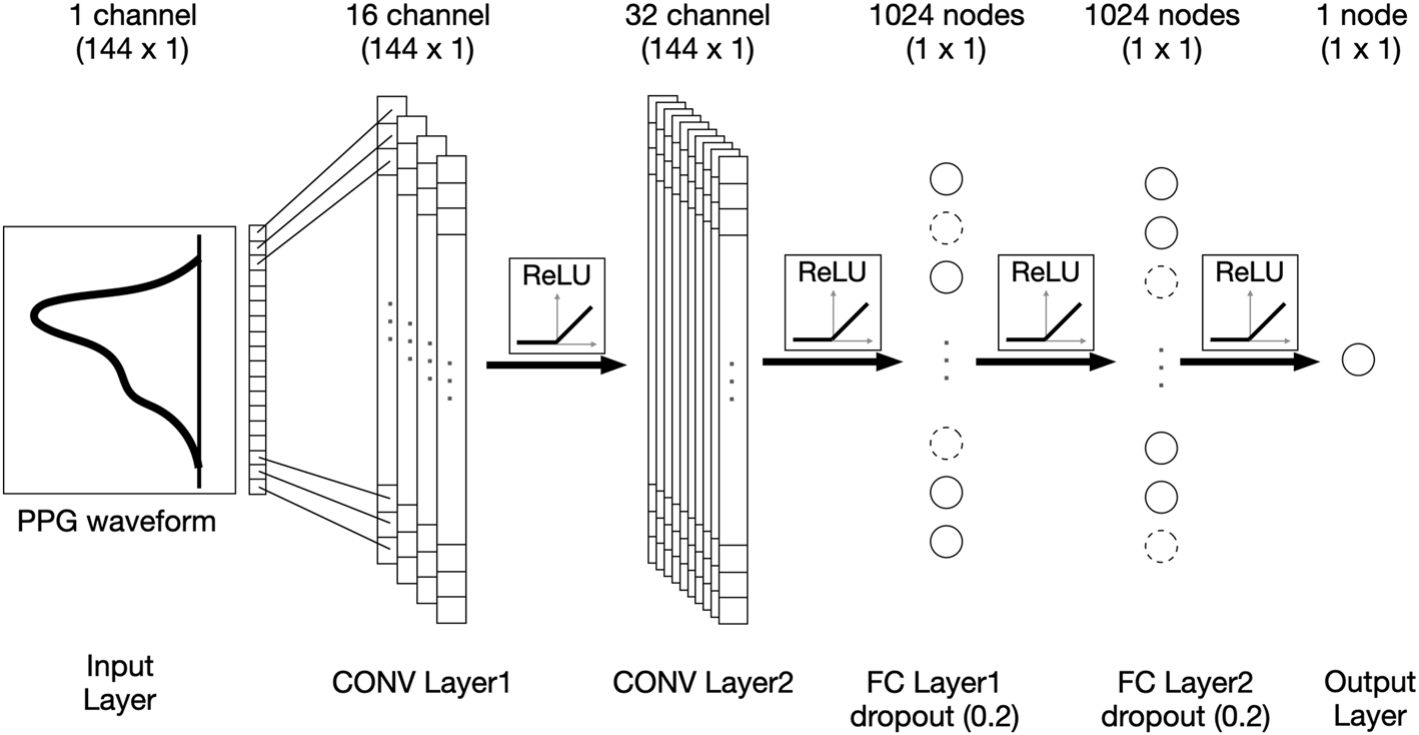
The structure of the developed model. The input layer was composed of a normalized photoplethysmogram with 144 × 1 matrices. The convolutional layers consisted of convolutional, non-linear layers with a different number of filters; 16 and 32 filters were employed for the first, second and third layer, respectively, which were of size 10 and 8 with a stride of 1. Subsequently, two fully connected layers were connected using 1024 nodes with a 0.2 dropout rate prior to the output neuron. PPG: photoplethysmogram, CONV: Convolution; FC: fully connected; ReLU: Rectified Linear Unit.

The structure of the convolutional neural network (CNN) employed in this study is a well-known architecture consisting of a CNN and a fully connected network, and its hyperparameters were heuristically chosen through the grid search method^35^. Table 2 shows that models with various structures or hyperparameters were created and evaluated to select the model with the best performance. The selected models are shown in bold in Table 2; and were validated using the train dataset for comparative evaluation with existing studies, considering that the performance of existing studies was evaluated with the data used for development without using a separate test set. The best CNN model was composed of two 1-dimensional convolutional layers, and two fully connected layers with 1024 nodes and 0.2 dropout rates prior to the output neuron. The input layer was composed of a normalized PPG. The PPG signals comprised 144 × 1 matrices, where ‘144’ is the number of time samples, and ‘1’ represents a single channel PPG. The convolutional layers consisted of convolutional, non-linear layers performing features extraction. For each convolutional layer, a different number of filters were employed; 16 and 32 filters were employed for the first, second and third layer, respectively, which were of size 10 and 8 with a stride of 1. As a non-linear activation function, the rectified linear unit (ReLU) function was employed in all layers. Figure 3 presents the structure of the developed model. The developed CNN was trained using a supervised learning approach by employing the mean squared error (MSE) as a loss function, and the Adam optimizer as an optimization algorithm. The Adam optimizer parameters were set to: 0.9 for the exponential decay rate of the moving average gradient (β1), and 0.999 for the exponential decay rate of the moving average of the squared gradient (β2). The optimization procedure was conducted with a batch size of 50 and was iterated until the validation accuracy was no longer improved within the next 100 iterations. In the validation process, a tenfold cross validation was used. At this time, the training and test sets were divided in a ratio of 9:1, and 25% of the training set were used as the validation set. Consequently, for each fold, data for 676 out of a total of 752 subjects was used as the training set, data for 76 subjects was used as the test set, and data for 507 subjects in the training set were used for training and 169 for validation. In the process of dividing the training, test, and validation sets, stratified data sampling was applied to evenly distribute the age for each dataset. The proposed CNN model was developed and validated using a 2.8 GHz Intel Core i9-10900F processor, 128 GB 1,600 MHz DDR3 RAM, NVIDIA Geforce™ RTX 3090, and Python 3.8: Anaconda, Tensorflow 2.5.

**Table 2:** Selected hyperparameters for the best performance model.

Hyperparameter	Compared options (Test)
Convolution layers number	**2** 3 4 5 6
Fully connected layers number	1 **2**
Number of nodes of FC	512 **1,024** 2,048
Dropout rate	0 0.1 **0.2** 0.3 0.4 0.5
Learning rate	0.001 **0.0001**
Activation function	ReLU
Optimizer	ADAM

Optimal values from Bayesian optimization are presented in bold

### Statistical validation

The age estimation performance of the developed deep learning model was quantitatively evaluated using the mean absolute error, root-mean-squared-error, Pearson’s correlation coefficient, coefficient of determination, and Bland–Altman analysis. Statistical analysis was performed on the entire data, and comparisons between groups according to hypertension, diabetes, drinking, and smoking, were also performed. To evaluate the significance between groups, an independent t-test was used for each group that satisfied the normality and homogeneity of variance, otherwise, the Mann–Whitney U test was used. The Shapiro–Wilk test was used to test the normality and the Levene’s test was used to test the homogeneity of variance. All of the data processing procedures and statistical verification processes were performed using Matlab 2020b (The MathWorks, Inc., MA, USA).

### Grad-CAM

A class activation map (CAM) is a method of determining the main elements that have a great influence on the classification result in an image by including global average pooling (GAP) in the last layer of the CNN model^36^. A CAM is a type of AI that can explain the inner workings of a deep-learning model that is considered a black box and can tell which element of the input data is important information for classification. However, to apply a CAM, a GAP layer must be added to the last layer of the model, resulting in a limitation when constructing a deep-learning model. A Grad-Cam is a method of calculating the weights that are connected to features by means of gradients, thereby allowing the CAM to be obtained without modifying the original deep-learning model structure^37^ (see Supplementary Material). Since a Grad-Cam can use the existing model structure as it is, it can be applicable to most CNN models, such as those that include a fully connected layer, a structured output, or multi-modal input.

### Ethical approval

The study protocols were approved (approval number: 2015 − 0104) and monitored by the institutional review board of the Asan Medical Center (Seoul, Republic of Korea), and informed consent to participate in the research studies was obtained from each subject before participating in the experiment. The entire research was performed in accordance with relevant guidelines and regulations.

## Results

### Performance of the developed model in aging assessment

**Figure 4:**
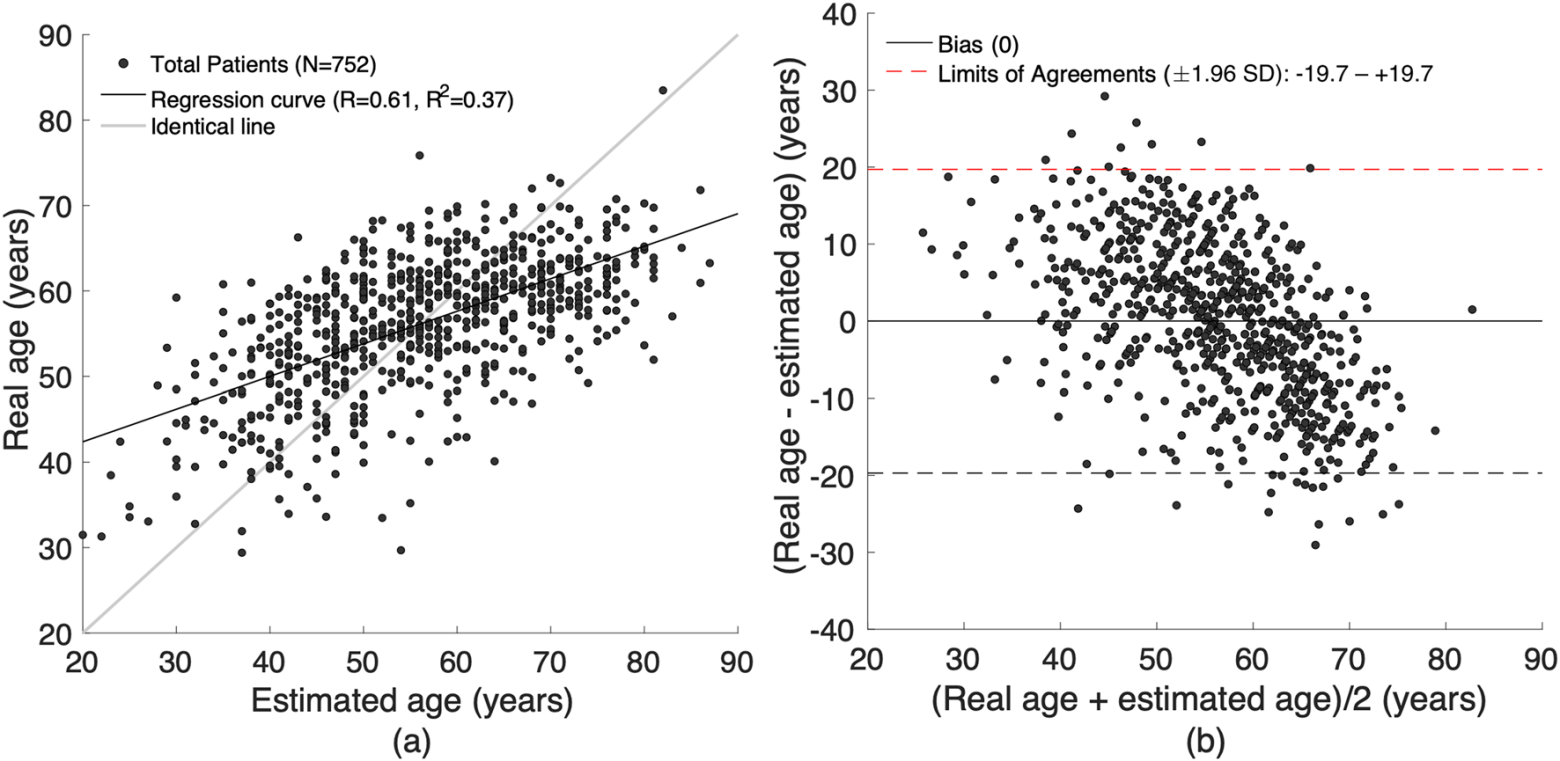
Plots for comparison between the estimated and actual ages of all subjects (N = 752). (**a**) Scattering plot, and (**b**) Bland–Altman plot. R: Pearson’s correlation coefficient; R^2^: Coefficient of determination; SD: standard deviation.

Table 3 shows the MAE, RMSE, range, correlation coefficient, and coefficient of determination of the age estimation using the developed model. The resultant values were calculated by compiling all the results of the test set obtained for each fold for cross validation. From the results, in the case of the total test data set, the developed model showed an MAE of approximately 8 years old and an RMSE of 10 years old in the 0.1–29.2 age bracket, with a correlation coefficient of 0.61, and a coefficient of determination of 0.37. Figure 4 shows the results of the Bland–Altman analysis. In the Bland–Altman plot, the bias can be seen to be approximately 0 for the entire range of 20–80 years, with most of the deviations in the estimated values and actual values being located within the 95% confidence interval. Here, the upper bound of the 95% confidence interval was 19.7, and the lower bound was approximately − 19.7. Moreover, there was no significant difference in the model performance based on hypertension, diabetes, drinking or smoking.

**Table 3:** Model performances.

Dataset	N	Model performance				
		MAE (years)	RMSE (years)	Error range (years)	R	R^2^
**Blood pressure**						
Hypertension	205	8.2	10.0	0.1 – 29.2	0.6	0.35
non-Hypertension	547	8.2	10.0	0 – 29.1	0.62	0.38
**Blood glucose**						
Diabetes mellitus	71	7.8	9.6	0.1 – 21.5	0.73	0.51
non-Diabetes mellitus	681	8.2	10.1	0 – 29.2	0.59	0.35
**Drinking**						
Alcohol	217	7.8	9.6	0 – 25.8	0.66	0.43
non-Alcohol	535	8.4	10.2	0 – 29.2	0.59	0.35
**Smoking**						
Smoking	109	8.9	10.8	0 – 25.8	0.6	0.36
non-Smoking	643	8.1	9.9	0 – 29.2	0.61	0.38
Total	752	8.1	10.0	0 – 29.2	0.61	0.37

### Grad-CAM

**Figure 5:**
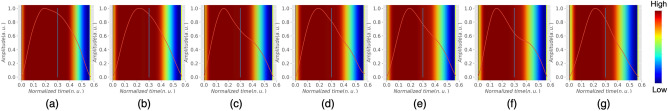
Photoplethysmogram waveform and Grad-Cam results for vascular aging assessment of subjects in their (**a**) 20 s, (**b**) 30 s, (**c**) 40 s, (**d**) 50 s, (**e**) 60 s, (**f**) 70 s, and (**g**) 80 s. Vertical line represents the midpoint of the time. With increased aging, the importance near the Pulse_onset_ decreases, and the interval of the intermediate level importance (yellow) in the falling phase tends to increase.

Figure 5 shows the PPG waveform and Grad-Cam results for each age group. In the figure, the red color represents a section with a greater weight, while the blue indicates a section with a smaller weight. The results of the Grad-Cam suggest that the waveform near the systolic peak plays the most important role in the assessment of vascular aging. These trends do not differ significantly across the age groups. However, if observed more closely, the importance of the waveform immediately after Pulse_onset_, and the overall area (red color) with high importance for aging assessment, decrease.

## Discussion

### Performance of the developed model for aging assessment

Table 4 shows the correlation coefficient, coefficient of determination, and RMSE of the model developed in this study, compared with the results of previous studies. The R value in this study was 0.61, which was similar to the R value of 0.63–0.66 from the existing feature-based SI and SDPPG studies. In addition, it has been reported that the developed model can estimate the age of blood vessels regardless of chronic diseases such as hypertension or diabetes mellitus, or lifestyles such as drinking and smoking. In this study, the number of convolutional layers, the number of fully connected layers, and the dropout rate, were adjusted for model optimization to develop a model and evaluate its performance. The average (range) of the correlation coefficients of all the models used was 0.59 ± 0.01 (0.55–0.61), and the average MAE (range) was 8.4 ± 0.1 years (8.2–8.6), confirming that there is a slight performance deviation between the models. However, the MAE estimation result shows that the difference in the performance according to the type of model is less than 1 year, suggesting that applying any one model will not have a significant effect on the age estimation result. The performances of the various models evaluated in the study are shown in Table 5. In addition, by intentionally overfitting the model, we examined the possibility of improving the performance of the proposed model according to data expansion. When the train and test data were not separated, it was confirmed that the R value of the proposed model was high (0.99) compared to the R value of in the LMO-based study evaluated based on 3 people (− 0.87 to − 0.98). Compared with the study using the PPG recorded using a smartphone, the R^2^ value was slightly lower than that of the feature-based study, however, the waveform-based study showed a higher performance, and the RMSE was lower than that in all the other cases. In summary, the performance of the model developed in this study showed a similar level of performance to that of the existing feature-based study, and with an improved performance compared with the study using waveforms. In addition, the developed model showed a very high correlation coefficient (R = 0.99) and coefficient of determination (R^2^ = 0.98) with the age in the training set, a low MAE of 1.5 years, and an RMSE of 2 years, which shows a large gap compared with the results of the test set. Although the results of the training set can be attributed to a larger inter-subject variation and overfitting, compared with the results of the existing regression model, it can be confirmed that the 1-D CNN-based model proposed in this study has a much better expressive power. In addition, since overfitting can generally be alleviated as the data increases, a performance improvement can be expected as the data expands. Another advantage of this study is that it uses the waveform as it is, without a separate feature extraction process. As such, compared to feature-based research, research using waveforms has the advantage of a lesser burden on feature point detection and reduction in errors due to feature point erroneous detection, which is advantageous in providing more accurate and stable results.

**Table 4:** Comparison of the correlation coefficient, coefficient of determination, and root-mean-squared error (RMSE) for various vascular aging assessment models.

Input type	Metric	N (age range)	Correlation coefficient	Coefficient of determination	RMSE (years)
Waveform	Proposed	752 (19–87)	0.61	0.37	10.0
	Resnet^21^	4,769 (18–79)	n.a	0.28	12.3
Derived Feature	Stiffness index^16^	87 (21–68)	0.65	n.a	n.a
	Stiffness index^29^	124 (20–74)	0.63	n.a	n.a
	Second derivative PPG aging index^24^	248 (< 60 yrs.)	− 0.37 (< 60 yrs.)	n.a	n.a
		276 (> 60 yrs.)	− 0.13 (> 60 yrs.)	n.a	n.a
	PPG augmentation index^24^	248 (< 60 yrs.)	0.21 (< 60 yrs.)	n.a	n.a
		276 (> 60 yrs.)	0.29 (> 60 yrs.)	n.a	n.a
	Second derivative PPG aging index^25^	600 (30–89)	0.80	n.a	n.a
	Second derivative PPG aging index^26^	93 (36–86)	0.30	n.a	n.a
	Ridge regression^21^	4,769 (18–79)	n.a	0.50	10.2
	Regression^21^	4,769 (18–79)	n.a	0.43	10.8
	Decomposed waveform feature ^18^	4 (13–39)	− 0.87–0.98	n.a	n.a
	Artificial Neural Network ^31^	757 (19–87)	0.63	n.a	10.0
	XGBoost ^32^	752 (19–87)	0.63	0.39	9.9

**Table 5:** Model performance according to the hyperparameters.

Evaluation metric	Mean ± SD (range)
Correlation coefficient (R)	0.59 ± 0.01 (0.55 – 0.61)
Coefficient of Determination (R^2^)	0.34 ± 0.02 (0.30 – 0.37)
Mean absolute error (years)	8.4 ± 0.1 (8.2 – 8.6)
Root-mean-squared error (years)	10.3 ± 0.2 (10.0 – 10.6)

### Grad-CAM

For the Grad-Cam results, it can be seen that the weight in the range including the PPG systolic and diastolic peaks, which is the center of the segment, is higher, and the weight at the edge near the Pulse_onset_ is lower. However, it cannot be concluded from this result alone that the pulse onset does not contribute to the estimation of vascular aging because the waveform near the pulse onset was cut off during the segmentation process. In addition, it can be intuitively confirmed that the systolic peak plays an important role in vascular aging, however, detailed verification through waveform decomposition or differential waveform is required to determine the contribution of the diastolic peak. It is presumed that the change in the Grad-Cam importance with increasing age may be caused by the change in the left ventricular contraction pattern or the reflected wave according to aging. In particular, the increase in the intermediate importance interval (yellow color) with increasing age may be related to the rapid return of the reflected wave.

## Conclusion

We proposed a method to assess vascular aging by applying deep learning to the PPG original waveform, and to identify which shape of the PPG waveform contributes to the assessment of vascular aging using XAI. In conclusion, although the feature detection process was omitted and only the original waveform itself was used, the performance of the proposed deep learning-based vascular aging assessment technique was comparable to that of the existing feature-based study and was higher than the original waveform-based study. This suggests that deep learning could automatically generate features from the PPG waveforms and have a higher significance over empirical and manually detected features in vascular aging assessment. The deep learning technique is expected to have a more improved performance in the assessment of vascular aging according to the data expansion. The results of this study not only suggest the possibility of assessing vascular aging using the PPG, but also provide implications for the principles and rationale of PPG-based vascular aging assessment through XAI-based result interpretation, which contribute to revealing the characteristics of vascular aging reflected in the PPG.

## Supplementary Information

Supplementary Information.

## Data Availability

The datasets generated and/or analyzed during the current study are not publicly available due to the scope of the data disclosure of the institutional review board but are available from the corresponding author upon a reasonable request.
