# Supplementary material for: Photoplethysmogram based vascular aging assessment using the deep convolutional neural network
Source: Sci Rep. 2022 Jul 5;12:11377. doi: 10.1038/s41598-022-15240-4 (PMC9256729; doi:10.1038/s41598-022-15240-4)
Supplement: Supplementary file 1 — Supplementary Information. [file 41598_2022_15240_MOESM1_ESM.pdf]

Grad-CAM heat-map calculation

To obtain the class-discriminative localization map, the Grad-CAM computes the gradient score for class c, y^c, with respect to feature maps, A, of the convolutional layer. The gradients flowing back are global-average-pooled to obtain the importance weights α_k^c:

$$\alpha_k^c = \frac{1}{N}\sum_i \sum_j \frac{\partial y^c}{\partial A_{i,j}^k}$$

where N is the total number of inputs. Similar to the CAM, the Grad-CAM heat-map, $L_{Grad-CAM}^c$, is a weighted combination of the feature maps, but is followed by a ReLU:

$$L_{Grad-CAM}^c = ReLU\left(\sum_k \alpha_k^c A^k\right)$$

If the architecture is already CAM compatible, the weights learned in the CAM are precisely equal to the weights computed in the Grad-CAM. Other than the ReLU, this makes the Grad-CAM a generalization of the CAM. This generalization is what allows the Grad-CAM to be applicable to any CNN-based architecture.
